# Diabetic Myonecrosis Presenting as Atraumatic Thigh Pain in a Patient With Poorly Controlled Type 1 Diabetes Mellitus

**DOI:** 10.51894/001c.165696

**Published:** 2026-07-31

**Authors:** C. Alex Matisse, Misbah Fatima, Anh Tuan Mai, Hassan Afzal Cheema

**Affiliations:** 1 DMC - Sinai Grace

## 100

### Introduction

Diabetes mellitus (DM) affects approximately 12% of the United States population. A rare complication of DM is diabetic myonecrosis (DMN), also known as diabetic muscle infarction, first described in 1965. Fewer than 200 cases have been reported, with an estimated incidence of less than 1% among diabetic patients. DMN typically occurs in patients with long-standing poorly controlled diabetes and often presents with acute atraumatic pain and swelling of the thigh or calf without systemic symptoms. The underlying mechanism is thought to involve diabetic microangiopathy leading to skeletal muscle ischemia and infarction, though the exact pathogenesis remains unclear. We present a case of diabetic myonecrosis in a male patient in his 30s with poorly controlled type 1 diabetes presenting with unilateral thigh pain.

### Case Presentation

A male in his 30s with type 1 DM, CKD stage 3a, hypertension, and HFpEF presented with two weeks of progressive left anterolateral thigh pain and swelling without trauma or systemic symptoms. Examination revealed a 1-cm superficial lesion and underlying 5 × 8 cm indurated, exquisitely tender area of the left thigh without crepitus or ecchymosis. Laboratory studies demonstrated glucose 319 mg/dL, creatinine 2.06 mg/dL (baseline 1.3 mg/dL), CPK 345 U/L, ESR 100 mm/hr, and HbA1c 15.6%. Prior imaging, including radiographs and Doppler ultrasound, was negative. Initial MRI showed diffuse muscle edema concerning for myositis. Despite treatment for suspected cellulitis and evaluation for autoimmune and infectious causes, symptoms persisted. Repeat MRI demonstrated diabetic myopathy with a 2.5 × 3.2 × 8 cm necrotic region in the vastus lateralis consistent with diabetic myonecrosis.

### Conclusion

Diabetic myonecrosis is an uncommon complication of poorly controlled diabetes that is frequently misdiagnosed as cellulitis, myositis, or deep vein thrombosis. MRI is the diagnostic modality of choice, typically demonstrating muscle edema with T2 hyperintensity. Recognition of this condition is important to avoid unnecessary invasive procedures and to initiate appropriate conservative management. This case highlights the importance of considering diabetic myonecrosis in patients with poorly controlled diabetes presenting with atraumatic limb pain.

